# Solitary AAH Arising from Extralobar Sequestration in A Less Than 3-year-old Boy: A Case Report

**DOI:** 10.3779/j.issn.1009-3419.2017.11.11

**Published:** 2017-11-20

**Authors:** Liqing LU, Chunfang ZHANG, Yuanda CHENG

**Affiliations:** Department of Thoracic Surgery, Xiangya Hospital, Central South University, Changsha 410008, China

**Keywords:** Atypical adenomatous hyperplasia, Pulmonary sequestration, Congenital diaphragmatic eventration, Child, Cancer predisposition syndrome

## Abstract

We present a case of two-year old boy with solitary atypical adenomatous hyperplasia (AAH) in extralobar sequestration (ELS), which was misdiagnosed as diaphragmatic hernia before surgery. Review of AAH and pulmonary sequestration (PS) revealed that the present case is the youngest of solitary AAH and also the first report of solitary AAH arising in ELS without a primary lung cancer. In a sense, the present case firstly supports the hypothesis that ELS may be an underlying cancer predisposition syndrome, so aggressive surgical therapy should be recommended for ELS.

## Introduction

Pulmonary sequestration (PS) consists 0.15%-6.4% of all congenital malformation of lung^[1]^. PS is often associated with other congenital anomalies, such as congenital diaphragmatic hernia (CDH) and congenital heart diseases. Atypical adenomatous hyperplasia (AAH) usually is associated with primary pulmonary adenocarcinoma. The review of the literatures reveals a few of cases of adenocarcinoma arising in PS, in which only one arising in extralobar sequestration (ELS)^[2]^, and solitary AAH had never been repoted in PS.

## Case presentation

A two-year-old boy was admitted to our department for left CDH indicated by chest X-ray, because of considering pulmonary infection in local hospital. Although the pulmonary infection was controlled by antibiotic therapy, the patient was still recommended to tertiary care center for further treatment of CDH. Electrocardiogram showed sinus tachycardia. Plain abdominal X-ray and barium meal revealed a prominent left hemidiaphragm and gastric bubble (Fig 1). Clinical diagnosis is CDH and left posterolateral thoracotomy with one-lung ventilation under general anesthesia was performed with the 6^th^ partial costectomy. During the operation, congenital diaphragmatic eventration (CDE) was confirmed instead of CDH and diaphragm replication was executed. In addition, ELS was detected unexpectedly and removed, considering the history of pulmonary infection. In operation, we found the blood supply of ELS arise from the abdominal aorta. To our surprise, postoperative pathology showed AAH of bronchiolo-alveolar epithelium in the resected lung parenchyma (Fig 2). The patient was discharged on the sixth postoperative day without any complications. Three years follow up was unremarkable, no radiological signs of recurrence.

**1:**
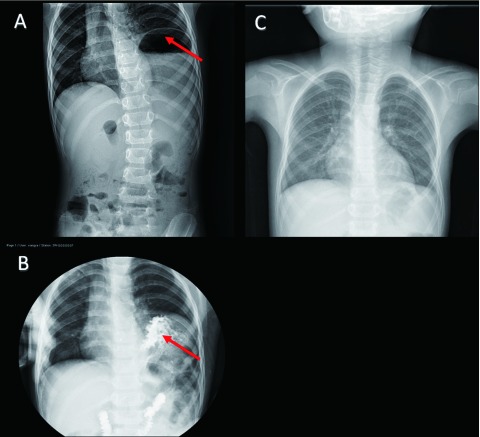
Preoperative plain abdominal radiograph upper gastrointestinal barium meal shows a raised left hemidiaphragm and gastric bubble (A & B). C is chest radiograph shows normal at follow-up of 3 years.

**2:**
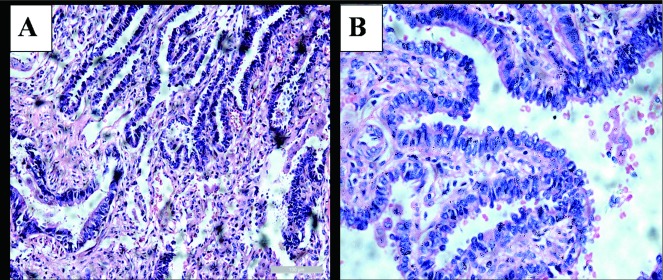
Histological examination of resected lung tissue demonstrates atypical adenomatous hyperplasia of bronchiolo-alveolar epithelium [H & E staining, ×200 (A) and ×400 (B)]

## Discussion

PS is a relatively rare congenital malformation, which comprises 0.15%-6.4% of all congenital pulmonary anomalies. Anatomically, PS includes two types: ELS which has its own pleural covering, and intralobular sequestration (ILS) which shares common pleura with the normal lung tissue^[1]^. ELS is more likely to be associated with other congenital anomalies than ILS, such as CDH or congenital heart disease^[2]^. In the present case, the patient was misdiagnosed as CDH before operation, but CDE was confirmed during surgery.

AAH is involved in the early stage of a complex multistep carcinogenesis of pulmonary adenocarcinoma and thought to be a probable forerunner^[3]^. The incidence of AAH has been reported to be about 9%-21% in primary lung cancer patients, while approximately 4%-10% in patients without lung cancer, and it is usually 0.5 cm or less and located in the peripheral area of the lung^[3]^. Previously reported cases of AAH in children occurred in combination with a congenital cystic adenomatoid malformation, or metastatic osteosarcoma of the lung^[4]^. To the best of our knowledge, this is the first report of solitary AAH arising in ELS without a primary lung cancer occurred in very early age. The youngest case of solitary AAH was a 12-year-old male in Korea, reported in 2016^[4]^. In addition our review of the literatures reveals less than ten cases of carcinoma arising in PS, including four cases of pulmonary adenocarcinoma, but only one arising in ELS^[5]^. Belchis *et al*^[5]^ hypothesized that the development of carcinoma in the sequestrations may be multifactorial and may include chronic inflammation and irritation. However, this hypothesis does not explain AAH in a patient under the age of three ages, who lacks a long-standing history of recurrent infection. Another hypothesis postulated that PS manifested as an underlying cancer predisposition syndrome and an innate propensity to undergo malignant degeneration. However, authors thought lack sufficient evidence, for all of the sequestration malignancies occurred in older patients^[5]^. In a sense, the present case, a two years old AAH patient, supports the hypothesis that ELS may be an underlying cancer predisposition syndrome.

Surgical resection is the conventional standard treatment for PS to prevent possible infection, congestive heart failure, and hemoptysis. However, if the symptoms of patients with PS are not serious, they are often left un-intervened. The management of ELS is more controversial, it is known that these lesions can remain asymptomatic throughout the patient's life but the complications may develop^[6]^. As previously mentioned, AAH is a forerunner of pulmonary adenocarcinoma. Without any treatment, AAH arises from ELS may worsen a patient's condition and even develop into adenocarcinoma. Although the incidence of AAH arising from ELS is low, its existence provided an argument for surgery. At least, patients should receive regular follow-up after treatment.

## Conclusion

We firstly reported the solitary AAH arising from ELS with CDE, and this AAH patient was the youngest in the world now. It is possible that AAH is one of the congenital anomalies associated with ELS, just like CDH and other congenital heart diseases, but the molecular mechanism is unclear. In our opinion, surgical treatment should be recommended in a patient with ELS regardless of their clinical symptoms since it might be an underlying cancer predisposition.
